# Disparities in Health Insurance Coverage and Health Status Among Farmworkers, Sonoma County, California, 2013–2014

**DOI:** 10.5888/pcd13.150519

**Published:** 2016-03-31

**Authors:** Kristin L. Moore, Jenny Mercado, Jana Hill, Sarah C. Katz

**Affiliations:** Author Affiliations: Jenny Mercado, Jana Hill, Sarah C. Katz, Health Policy, Planning, and Evaluation Division, County of Sonoma Department of Health Services, Santa Rosa, California.

## Abstract

**Introduction:**

The Sonoma County Farmworker Health Survey (FHS) was conducted to describe the health and well-being of adult farmworkers in Sonoma County, California, and to identify preventable health disparities for this population.

**Methods:**

From September 2013 through January 2014, venue-based and convenience sampling were used to survey 293 farmworkers aged 18 years or older. The questions included self-rated general health, diabetes and hypertension, and body mass index. To identify disparities between surveyed farmworkers and Sonoma County residents overall, age-adjusted prevalence estimates were developed by using indirect standardization to the adult (≥18 years) Sonoma County sample from the California Health Interview Survey for 2011–2012.

**Results:**

Surveyed farmworkers were mostly male (91%) and Latino or Hispanic (95%), and 54% had an educational attainment of 8th grade or less. Most (81%) farmworkers reported their families earned less than $30,000 in 2012. After adjusting for age, 30% of farmworkers had US-based health insurance as compared with the 86% of Sonoma County adults in 2011–2012 (*P* < .001), and 15% of farmworkers reported ever being diagnosed with diabetes after adjusting for age as compared with 5% of Sonoma County adults (*P* = .002). After adjusting for age, 44% of farmworkers reported poor or fair health in general as compared with 13% of Sonoma County adults (*P* < .001).

**Conclusion:**

We identified significant health disparities between Sonoma County farmworkers and Sonoma County adults overall. Additional research and new health policies are necessary to eliminate these health disparities and to facilitate farmworker access to the health care system.

**MEDSCAPE CME**

Medscape, LLC is pleased to provide online continuing medical education (CME) for this journal article, allowing clinicians the opportunity to earn CME credit.

This activity has been planned and implemented in accordance with the Essential Areas and Policies of the Accreditation Council for Continuing Medical Education through the joint providership of Medscape, LLC and *Preventing Chronic Disease*. Medscape, LLC is accredited by the ACCME to provide continuing medical education for physicians.

Medscape, LLC designates this Journal-based CME activity for a maximum of 1 ***AMA PRA Category 1 Credit(s)™***. Physicians should claim only the credit commensurate with the extent of their participation in the activity.

All other clinicians completing this activity will be issued a certificate of participation. To participate in this journal CME activity: (1) review the learning objectives and author disclosures; (2) study the education content; (3) take the post-test with a 75% minimum passing score and complete the evaluation at http://www.medscape.org/journal/pcd; (4) view/print certificate.

**Release date: March 31, 2016; Expiration date: March 31, 2017**

**Learning Objectives**

Upon completion of this activity, participants will be able to:

1. Distinguish health insurance disparities among adult farmworkers in Sonoma County, California, based on a cross-sectional study
2. Identify disparities in high blood pressure, diabetes, and obesity among adult farmworkers in Sonoma County, California
3. Determine disparities in self-reported health status among adult farmworkers in Sonoma County, California

**EDITOR**

Caran Wilbanks

Editor, Preventing Chronic Disease

Disclosure: Caran Wilbanks has disclosed no relevant financial relationships.

**CME AUTHOR**

Laurie Barclay, MD

Freelance writer and reviewer, Medscape, LLC

Disclosure: Laurie Barclay, MD, has disclosed the following relevant financial relationships:

Owns stock, stock options, or bonds from: Pfizer

**AUTHORS**

Kristin L. Moore, MPH, Health Policy, Planning, and Evaluation Division, County of Sonoma Department of Health Services, Santa Rosa, California

Disclosure: Kristin L. Moore, MPH, has disclosed no relevant financial relationships.

 Jenny Mercado, MPH, Health Policy, Planning, and Evaluation Division, County of Sonoma Department of Health Services, Santa Rosa, California

Disclosure: Jenny Mercado, MPH, has disclosed no relevant financial relationships.

 Jana Hill, MPH, Health Policy, Planning, and Evaluation Division, County of Sonoma Department of Health Services, Santa Rosa, California

Disclosure: Jana Hill, MPH, has disclosed no relevant financial relationships.

 Sarah C. Katz, MPH, Health Policy, Planning, and Evaluation Division, County of Sonoma Department of Health Services, Santa Rosa, California

Disclosure: Sarah C. Katz, MPH, has disclosed no relevant financial relationships.

## Introduction

Significant differences in health outcomes, particularly among individuals in different racial, ethnic, and socioeconomic groups, have been widely acknowledged (1,2). These health disparities among minority populations account for higher premature death and infant mortality rates, higher disease burden, and poorer access to health care when compared with the US national average (3). As the United States continues to experience major demographic shifts, health disparities and their causal conditions must be reduced or eliminated if the country is to continue health improvements. Reducing disparities has been prioritized by the US Surgeon General, the Institute of Medicine, the Centers for Disease Control and Prevention (CDC), and other leading health and public health agencies (1,4).

The 1999 California Agricultural Workers Health Survey (CAWHS) was a study of farmworker health in California. The study found that California farmworkers were mostly Hispanic/Latino men and undocumented immigrants and had poor access to medical care. Nearly 20% of male farmworkers had at least 2 major risk factors (high serum cholesterol, high blood pressure, or obesity) for chronic disease (5). This research highlights a minority population that has poor access to health care and health outcomes. However, these data were not compared with the overall California population or with the California immigrant population to better understand disparities in health behaviors and health outcomes.

In Sonoma County, California, agriculture is a major contributor to the local economy, and agricultural land use accounts for over 60% of the county’s acreage. A healthy agricultural workforce is critical to maintaining the local economy; yet, before our study no data on the health and well-being of local farmworkers were available. To bridge this gap, the Sonoma County Farmworker Health Survey (FHS) was developed with the primary goal of describing the health and well-being of adult farmworkers in Sonoma County and identifying preventable health and wellness disparities. On the basis of prior state and national research, we hypothesized that Sonoma County farmworkers would have less health care access and greater chronic disease prevalence than the general adult Sonoma County population.

## Methods

The FHS was a cross-sectional study of farmworkers in Sonoma County, California, conducted from September 2013 through January 2014. Farmworkers that spoke either English or Spanish, were 18 years of age or older, and had completed any farm work in the preceding 12 months were eligible to participate. A key objective of this study was to describe access to health care and the prevalence of preventable health conditions. To achieve this goal, sample size calculations were based on research examining health insurance status, high blood pressure, and obesity among farmworkers in California (5). Venue-based and convenience sampling were used to survey farmworkers from September 2013 through January 2014. Venue-based sampling was conducted at key sites where farmworkers were located, including day labor centers, community health clinics, and farms throughout Sonoma County. Among the 29 venues approached to participate, 18 (62% participation rate) agreed to allow data collection. Of the 11 venues that declined to participate, 3 sites were dairies, 4 were farms growing grapes for wine, 2 were other types of farms, and 2 were labor management companies.

The FHS instrument included 130 questions related to health and well-being. For data in this report, we used valid and reliable questions from the California Health Interview Survey (CHIS), a California state-wide survey of health and health care needs (6). To determine self-rated health status, participants in both CHIS and FHS were asked, “Would you say that in general your health is excellent, very good, good, fair, or poor?” Participants were also asked if a doctor had ever diagnosed them with diabetes or high blood pressure, and they were asked if they had US-based health insurance. Participants were asked about their height without shoes and weight when not pregnant to calculate body mass index (BMI). BMIs of 25.0 or more were categorized as overweight or obese, consistent with CDC guidelines (7). Data on demographic variables associated with social determinants of health were collected, including data on sex, ethnicity, income, educational attainment, English speaking ability, and family structure. Farmworkers were not asked about their immigration status in the United States. Participants were also asked to self-define if they were farmworkers full-time, part-time, or seasonally and the type of crop at their current or previous farm work job during the previous 12 months.

The FHS survey instrument was available in English and Spanish. From September 2013 through January 2014, 300 farmworkers were surveyed. The survey was administered in-person by trained bilingual and bicultural interviewers. All interview participants were given a $10 gift card and a packet with local health care and other resources on completion of the interview. This protocol was approved by Western Institutional Review Board.

Data for 7 farmworkers were excluded because of missing or unreliable responses; analyses were conducted on responses from 293 farmworkers using SAS statistical software (SAS Institute Inc). Descriptive statistics were calculated to describe the study population. To identify health disparities between surveyed farmworkers and Sonoma County residents overall, age-adjusted prevalence estimates were developed using indirect standardization to the adult (≥18 years) Sonoma County sample from the CHIS for 2011–2012. Age-adjusted estimates included prevalence estimates, 95% confidence intervals (CIs), and *P* values. The relationship between self-reported health status and diabetes, high blood pressure, and obesity was assessed to determine if poor or fair health in general was correlated with poorer self-reported health outcomes among farmworkers. Bivariate statistics were calculated using χ^2^ tests. Multivariable regression models were not developed because of a lack of statistical power. Significance was set at *P* < .05.

## Results

Of the 293 farmworkers surveyed, most (91.1%) were male, Latino or Hispanic (95.4%), and of Mexican (89.9%) ancestry (Table 1). Most (68.6%) farmworkers were aged 18 to 44 years (median, 37 years). Educational attainment among farmworkers was low, with 54% of farmworkers having an educational attainment of 8th grade or less. Of 182 farmworkers who remembered and reported their total family income in 2012, 148 (81.3%) reported earning less than $30,000. Three-quarters of farmworkers were linguistically isolated, with 73.0% reporting speaking English not at all or a little. Of those reporting family structure, 43.3% of farmworkers reported being married or living with a partner and children, 32.5% reported being single, and 24.2% were married or living with a partner. Nearly all (88.3%) of surveyed farmworkers considered Sonoma County their permanent residence, and 91.5% reported wine grapes as the primary crop in their current or most recent agricultural position. Farmworkers reported being full-time farmworkers who worked for the owner or grower (42.1%), being full-time farmworkers who worked for a contractor or a labor management company or not knowing who their boss was (32.5%), or being part-time or seasonal farmworkers (25.3%).

**Table 1:** Demographic Characteristics of Surveyed Adult Farmworkers (n = 293), Sonoma County Farmworker Health Survey, 2013–2014

Characteristic^a^	n (%)^b^
**Sex**	
Male	267 (91.1)
Female	26 (8.9)
**Median (minimum–maximum) age, y**	37 (18–75)
**Age group, y**	
18–24	39 (13.6)
25–34	89 (31.0)
35–44	69 (24.0)
45–54	51 (17.8)
≥55	39 (13.6)
**Latino or Hispanic ancestry**	
Latino or Hispanic	272 (95.4)
Non-Latino or non-Hispanic	13 (4.6)
**Region of ancestry**	
Mexican	250 (89.9)
Mexican American	15 (5.4)
Central American	8 (2.9)
Other	5 (1.8)
**Total family income in 2012, $**	
≤9,999	26 (9.4)
10,000–19,999	49 (17.6)
20,000–29,999	73 (26.3)
≥30,000	34 (12.2)
Don’t remember	96 (34.5)
**Highest educational attainment**	
8th grade or less	150 (54.0)
9th grade through high school graduate or equivalent	117 (42.1)
More than high school graduate	11 (4.0)
**English speaking ability**	
Not at all or a little	208 (73.0)
Somewhat, well, or very well	77 (27.0)
**Farmworker family structure**	
Single	94 (32.5)
Married/living with partner	70 (24.2)
Married/living with partner and child(ren)	125 (43.3)
**Permanent resident of Sonoma County**	
Yes	250 (88.3)
No	33 (11.6)
**Crop**	
Grapes	268 (91.5)
Other crops, not grapes	25 (8.5)
**Category of farmworker**	
Full-time farmworker working for owner or grower	123 (42.1)
Full-time farmworker working for contractor or labor management company or farmworker didn’t know who boss was	95 (32.5)
Part-time or seasonal farmworker	74 (25.3)

a Unless otherwise specified, data are presented as n (%).

b Estimates with less than 10% missing responses were included in analysis. Because of missing values, denominators may not add up to 293 for each variable. Percentages may not add up to 100% because of rounding.

After adjusting for age, 29.6% of farmworkers had US-based health insurance as compared with 85.7% of Sonoma County adults who had health insurance (*P* < .001) (Table 2). Farmworkers with US-based health insurance were significantly more likely to be female (*P* = .003), married or living with their partner and children (*P* = .03), and to have higher educational attainment (*P* = .048) (Table 3). Although not significant at *P* < .05, farmworkers doing farm work full-time where their current or previous boss was the owner or grower were more likely than other farmworkers to have US-based health insurance (*P* = .07). Also, although not significant at *P* < .05, farmworkers aged 25 to 34 years were less likely to have US-based health insurance than those in other age groups (*P* = .06) (Table 3).

**Table 2:** Age-Adjusted Prevalence of US-Based Health Insurance and Health Characteristics Among Surveyed Adult Sonoma County Farmworkers (n = 293), 2013–2014, and Adults Overall in Sonoma County, 2011–2012

Status	Sonoma County Farmworkers^a^		Sonoma County Adults^b^		*P* Value
	n	Age-Adjusted % (95% CI)^c^	Weighted n	% (95% CI)	
Health insurance	81	29.6 (23.2–36.1)	53,000	85.7 (81.1–90.4)	<.001
Self-rated general health as poor or fair^d^	115	43.9 (35.9–51.9)	49,000	13.1 (9.4–16.8)	<.001
Overweight or obese^e^	201	93.8 (80.8–106.7)	80,000	54.3 (48.4–60.3)	<.001
Ever diagnosed with diabetes	23	14.5 (8.6–20.4)	19,000	5.2 (3.0–7.4)	.002
Ever diagnosed with high blood pressure	49	26.0 (18.6–33.3)	93,000	24.8 (20.2–29.3)	.75

Abbreviation: CI, confidence interval.

a Data from Sonoma County Farmworkers Health Survey 2013–2014.

b Data from Sonoma County adult California Health Interview Survey 2011–2012.

c Indirect standardization to California Health Interview Survey population was used to develop age-adjusted prevalence.

d Farmworkers were asked to rate their health in general as excellent, very good, good, fair, or poor.

e Overweight or obese was defined as a body mass index (BMI) of 25.0 kg/m^2^ or more based on self-reported height and weight. Data on height and weight were missing for 21% of respondents, so estimates should be interpreted with caution.

**Table 3:** US-Based Health Insurance Status and Self-Reported General Health by Selected Demographics and Health Outcomes, Sonoma County Farmworker Health Survey (n = 293), 2013–2014

Characteristic	Health Insurance Status			Self-Rated Health Status^a^		
	No Health Insurance, n (Row %)^b^	Health Insurance, n (Row %)^b^	*P* Value^c^	Poor or Fair, n (Row %)^b^	Excellent, Very Good, or Good, n (Row %)^b^	*P* Value^c^
**Total^d^**	206 (71.5)	82 (28.5)	NA	117 (40.2)	174 (59.8)	NA
**Sex**						
Male	194 (74.1)	68 (26.0)	.003	104 (39.3)	161 (60.8)	.29
Female	12 (46.2)	14 (53.9)		13 (50.0)	13 (50.0)	
**Age, y**						
18–24	26 (66.7)	13 (33.3)	.06	13 (33.3)	26 (66.7)	.002
25–34	73 (83.0)	15 (17.1)		24 (27.3)	64 (72.7)	
35–44	46 (67.7)	22 (32.4)		34 (49.3)	35 (50.7)	
45–54	31 (62.0)	19 (38.0)		20 (39.2)	31 (60.8)	
≥55	25 (67.6)	12 (32.4)		24 (61.5)	15 (38.5)	
**Total family income in 2012, $**						
≤9,999	15 (65.2)	8 (34.8)	.11	14 (53.9)	12 (46.2)	.33
10,000–19,999	39 (79.6)	10 (20.4)		19 (38.8)	30 (61.2)	
20,000–29,999	55 (75.3)	18 (24.7)		26 (35.6)	47 (64.4)	
≥30,000	19 (55.9)	15 (44.1)		11 (33.3)	22 (66.7)	
Don’t remember	73 (76.0)	23 (24.0)		44 (46.3)	51 (53.7)	
**Highest educational attainment**						
8th grade or less	112 (76.2)	35 (23.8)	.048	63 (42.3)	86 (57.7)	.62
9th grade or more	83 (65.4)	44 (34.7)		50 (39.4)	77 (60.6)	
**Farmworker family structure**						
Single	73 (80.2)	18 (19.8)	.03	36 (38.3)	58 (61.7)	.92
Married/living with partner	51 (75.0)	17 (25.0)		28 (40.0)	42 (60.0)	
Married/living with partner and child(ren)	80 (64.0)	45 (36.0)		51 (41.1)	73 (58.9)	
**Permanent resident of Sonoma County**						
No	24 (75.0)	8 (25.0)	.56	12 (36.4)	21 (63.6)	.63
Yes	173 (70.0)	74 (30.0)		102 (40.8)	148 (59.2)	
**Category of farmworker**						
Full-time farmworker working for owner or grower	79 (64.8)	43 (35.3)	.07	—e		
Full-time farmworker working for contractor or labor management company or farmworker didn’t know who boss was	70 (76.1)	22 (23.9)				
Part-time or seasonal farmworker	57 (78.1)	16 (21.9)				
**Ever been diagnosed with high blood pressure**						
No	—f			75 (31.4)	164 (68.6)	<.001
Yes				39 (79.6)	10 (20.4)	
**Ever been diagnosed with diabetes**						
No	—f			96 (36.6)	166 (63.4)	.001
Yes				18 (72.0)	7 (28.0)	

Abbreviation: NA, not applicable.

a Self-rated general health as excellent, very good, good, fair, or poor.

b Row % = row percentages shown. Percentages might not add to 100 because of rounding.

c χ^2^ *P* value.

d Estimates with less than 10% missing responses were included in analysis. Because of missing values, denominators may not add up to the total for each column.

e Bivariate analyses for category of farmworker was not conducted because variable was not hypothesized to be a predictor of self-reported general health.

f Bivariate analyses for high blood pressure and diabetes were not conducted because variables were not hypothesized to be predictors of health insurance.

No disparity in age-adjusted prevalence of high blood pressure diagnoses between farmworkers and Sonoma County adults (26.0% and 24.8%, respectively) was found. After adjusting for age, 14.5% of farmworkers reported ever being diagnosed with diabetes as compared with 5.2% of Sonoma County adults (*P* = .002). After adjusting for age, 93.8% of farmworkers were categorized as overweight or obese as compared with 54.3% of Sonoma County adults (*P* < .001) (Table 2). However, BMI data were missing for 21% of farmworkers, so results should be interpreted with caution.

After adjusting for age, 43.9% of farmworkers reported poor or fair health in general as compared with 13.1% of Sonoma County adults (*P* < .001) (Table 2). Farmworker age was significantly associated with self-reported health status. Farmworkers 55 years or older were the most likely to report poor or fair health in general (*P* = .002) (Table 3). Farmworker sex, total family income in 2012, and family structure were not associated with self-reported health status. Farmworkers ever diagnosed with diabetes were significantly more likely to report poor or fair health in general when compared with farmworkers without diabetes (*P* = .001). Similarly, farmworkers ever diagnosed with high blood pressure were significantly more likely to report poor or fair health in general when compared with farmworkers never diagnosed with high blood pressure (*P* < .001) (Table 3).

## Discussion

To our knowledge, this is the first study identifying health disparities between a local farmworker population and the general population. This study of adult Sonoma County farmworkers found limited US-based health insurance coverage among farmworkers and disparities in health insurance status. Health insurance facilitates access to the medical system and safeguards against the cost of catastrophic illness (8). People with insurance are more likely to obtain health screenings, to receive care for chronic conditions, and to receive high-quality medical care and are less likely to have undiagnosed conditions (8–10). Among farmworkers especially, health insurance plays a critical role in the farmworker’s decision to seek medical treatment (11).

The demographics and prevalence of the uninsured has changed since the Patient Protection and Affordable Care Act (PPACA) was passed in 2010; yet, health insurance options for undocumented immigrants are largely unchanged, because adult undocumented immigrants cannot enroll in Medicaid or Medicare and are ineligible for tax credits or subsidies (12). Immigration status was not measured in FHS, but data from the 2012 National Agricultural Workers Survey (NAWS) indicate that most California farmworkers are undocumented immigrants (13). Undocumented immigrant farmworkers may be the most likely to delay needed medical treatment (11), so more research is needed to explore the relationship between immigration status, low health insurance coverage, and use of health care among Sonoma County farmworkers. Most FHS participants were men, and men in Sonoma County are less likely to have health insurance than women (14). More research involving multivariable regression models is needed to determine if the disparity in health insurance was confounded by sex.

Although not significant at *P* < .05, our finding was that farmworkers who self-defined as working full-time for a farm grower or owner were more likely to have health insurance than were farmworkers who self-defined as working full-time for labor management companies or labor contractors. This result is supported by a study finding that farmworkers working for growers or owners were more willing to seek treatment of work-related injuries than were farmworkers working for labor contractors. It was hypothesized that farmworkers working for owners were provided employer-based health insurance while farmworkers working for contractors were not, leading to farmworkers employed by the owner being more willing to seek medical treatment (11). PPACA employer mandates require that employers of more than 50 employees provide health insurance (15); more research is needed to understand farmworker employer’s compliance with this mandate.

Surveyed Sonoma County farmworkers were more than 3 times as likely to report poor or fair health in general as Sonoma County adults, suggesting health disparities between farmworkers and the general population. An individual’s own appraisal of his or her general health status is a powerful predictor of future illness and death (16). We found that farmworkers reporting poor or fair health in general were significantly more likely to be obese or to have ever been diagnosed with high blood pressure or diabetes, suggesting that self-reported health is a good summary measure of health status among farmworkers.

Prevalence of overweight or obesity was significantly higher among farmworkers than among Sonoma County adults overall, a result consistent with CAWHS, which found that 79% of male farmworkers and 74% of female farmworkers were overweight or obese (5). Excess body weight has far-reaching implications for health, including increasing risk of type 2 diabetes mellitus, cardiovascular disease, cancer, and premature death (17). However, 21% of BMI data was missing from the FHS analysis, so these results require further research. This large percentage of missing BMI data also suggest that self-reported height and weight measurements may be an inaccurate method of gathering BMI data from farmworkers.

The prevalence of diabetes among farmworkers was 3 times higher than that among Sonoma County adults. Access to medical care, medications, and diabetes self-management education are critical to the care of diabetes and are necessary to improve patient outcomes (18). Uninsured adults with diabetes are mostly from low-income racial/ethnic minority populations, and these individuals are less likely to access needed medical care for diabetes control (19–21). Given that farmworkers had a high prevalence of diabetes, most farmworkers were uninsured, and nearly all farmworkers had low incomes, the Sonoma County farmworker population should be prioritized for further diabetes research and prevention efforts. Undiagnosed diabetes is also most prevalent among the uninsured (20), suggesting that there may be farmworkers with undiagnosed diabetes in Sonoma County and that outreach efforts should include diabetes screening. Similarly, while no disparity in high blood pressure diagnosis was observed between Sonoma County farmworkers and the general population, more information is needed to determine if the lack of disparity was due to a high prevalence of undiagnosed high blood pressure among farmworkers.

Our results should be interpreted in the context of several limitations. This applied research study used nonrandom sampling of participant farmworkers, which may limit the generalizability of results. However, demographics of farmworkers in this report are similar to demographics of California farmworkers in the 2012 NAWS (13), suggesting that the FHS sample may be representative. The venue participation rate in FHS was low (62%), which may also limit generalizability. Both labor management companies that were approached to participate declined, so the responses of farmworkers working for labor management companies may be underrepresented. Use of other methods for sampling farmworkers, such as household-based sampling, could increase the response rate of future studies. Immigration status was not directly asked in this study because of sensitivity of the topic. Self-reported health outcomes were not confirmed by health examinations. Sample sizes for the Sonoma County adult sample were too small to allow for comparison of farmworkers with Latino/Hispanic men in Sonoma County overall. This survey included only farmworkers that had completed any farm work in the previous 12 months, so farmworkers that were too ill to work during this timeframe were excluded, potentially making this sample of farmworkers healthier than the true farmworker population.

We found that farmworkers had significantly poorer health access and health outcomes than did Sonoma County adults overall. These findings have been widely disseminated to policy makers and stakeholders in an effort to raise awareness about disparities between farmworkers and the overall local population and to prioritize farmworkers for future health policies and interventions. Stakeholders have begun using these results to support funding efforts and to define the policies needed to address the disparities. It is hypothesized that most Sonoma County farmworkers are undocumented immigrants, so these undocumented farmworkers will be ineligible for expanded health insurance under PPACA. As such, health care access will remain a barrier to improving poor health status and reducing the burden of chronic disease. In California overall, political will for providing health care coverage to undocumented immigrants may be increasing. Senate Bill 4 provides Medicaid coverage to undocumented children aged less than 19 years starting in May 2016 (22), and this bill is a step toward eliminating poor access to health care among undocumented immigrant farmworkers in California. In Sonoma County, ongoing research aims to clearly describe the relationship between health insurance, health care access and use, and immigration status among adults. This research involves obtaining de-identified data from Sonoma County federally qualified health centers (FQHCs), which will be used to identify current health care use among undocumented immigrants at FQHCs and any disparities in use. Additionally, a survey of adult undocumented immigrants is being conducted with the goal of describing health care access and use and barriers to accessing health care in Sonoma County. Results from FHS and this research will be essential when developing policies to improve access to health care among undocumented adult immigrants, including farmworkers.

## Figures and Tables

**Table:** Evaluation

**1. The activity supported the learning objectives.**				
**Strongly Disagree**	** **	** **	** **	**Strongly Agree**
1	2	3	4	5
**2. The material was organized clearly for learning to occur.**				
**Strongly Disagree**	** **	** **	** **	**Strongly Agree**
1	2	3	4	5
**3. The content learned from this activity will impact my practice.**				
**Strongly Disagree**	** **	** **	** **	**Strongly Agree**
1	2	3	4	5
**4. The activity was presented objectively and free of commercial bias.**				
**Strongly Disagree**	** **	** **	** **	**Strongly Agree**
1	2	3	4	5

## Post-Test Information

To obtain credit, you should first read the journal article. After reading the article, you should be able to answer the following, related, multiple-choice questions. To complete the questions (with a minimum 75% passing score) and earn continuing medical education (CME) credit, please go to http://www.medscape.org/journal/pcd. Credit cannot be obtained for tests completed on paper, although you may use the worksheet below to keep a record of your answers. You must be a registered user on Medscape.org. If you are not registered on Medscape.org, please click on the “Register” link on the right hand side of the website to register. Only one answer is correct for each question. Once you successfully answer all post-test questions you will be able to view and/or print your certificate. For questions regarding the content of this activity, contact the accredited provider, CME@medscape.net. For technical assistance, contact CME@webmd.net. American Medical Association’s Physician’s Recognition Award (AMA PRA) credits are accepted in the US as evidence of participation in CME activities. For further information on this award, please refer to http://www.ama-assn.org/ama/pub/about-ama/awards/ama-physicians-recognition-award.page. The AMA has determined that physicians not licensed in the US who participate in this CME activity are eligible for ***AMA PRA Category 1 Credits™***. Through agreements that the AMA has made with agencies in some countries, AMA PRA credit may be acceptable as evidence of participation in CME activities. If you are not licensed in the US, please complete the questions online, print the AMA PRA CME credit certificate and present it to your national medical association for review.

## Post-Test Questions

### Study Title: Disparities in Health Insurance Coverage and Health Status Among Farmworkers, Sonoma County, California, 2013–2014

#### CME Questions

1. You are advising a health maintenance organization in Sonoma County, California, regarding healthcare disparities among adult farmworkers. According to the cross-sectional study by Moore and colleagues, which of the following statements about health insurance disparities is ***correct***?
  - An estimated 30% of farmworkers and 86% of Sonoma County adults in 2011-2012 had health insurance
  - After adjustment for age, the difference in health insurance between farmworkers and Sonoma County adults was not statistically significant
  - Farmworkers with health insurance were significantly more likely to be men and living alone
  - Farmworkers working full time for a farm grower or owner were significantly less likely to have health insurance vs those working full time for labor management companies or contractors
2. According to the cross-sectional study by Moore and colleagues, which of the following statements about disparities in high blood pressure, diabetes, and obesity among adult farmworkers in Sonoma County is ***correct***?
  - High blood pressure was significantly more common in farmworkers than in Sonoma County adults
  - An estimated 15% of farmworkers reported ever being diagnosed with diabetes, after accounting for age, vs 5% of Sonoma County adults (*P *=.002)
  - After adjustment for age, 44% of farmworkers were categorized as overweight or obese, vs 24% of Sonoma County adults (*P *<.0001)
  - Undiagnosed diabetes or hypertension was unlikely to affect the results
3. According to the cross-sectional study by Moore and colleagues, which of the following statements about disparities in self-reported health status among adult farmworkers in Sonoma County, California, would ***most*** likely be accurate?
  - After adjustment for age, 44% of farmworkers reported poor or fair health in general, vs 13% of Sonoma County adults (*P *<.001)
  - Farmworker age was not significantly associated with self-reported health status
  - Farmworker gender, total family income, and family structure were significantly associated with self-reported health status
  - Self-reported health is a poor summary measure of health status among farmworkers
